# The impact of postpartum psychosis on partners

**DOI:** 10.1186/s12884-018-2055-z

**Published:** 2018-10-23

**Authors:** Nia Holford, Sue Channon, Jessica Heron, Ian Jones

**Affiliations:** 1 South Wales Clinical Psychology Doctorate Programme, Cardiff University, Cardiff, UK; 2 Centre for Trials Research, Cardiff University, Cardiff, UK; 3 Action on Postpartum Psychosis, Department of Psychiatry, The Barberry National Centre for Mental Health, University of Birmingham, Birmingham, UK; 4 National Centre for Mental Health, Division of Psychological Medicine and Clinical Neurosciences, MRC Centre for Neuropsychiatric Genetics and Genomics, Cardiff University, Cardiff, UK

**Keywords:** Postpartum Psychosis, Partners, Experience, Loss, Support, Relationship

## Abstract

**Background:**

Postpartum Psychosis is a severe mental health condition following childbirth, with a psychosis and associated mood disturbance. Research to date has primarily focused on mothers’ experiences, and on identifying risk factors, aetiology, and intervention efficacy. Within both research and clinical communities, there has been little acknowledgement of partners’ experiences of Postpartum Psychosis, nor the important support role that partners can provide. The aim of this study was to consider the lived experiences of partners of women who have had Postpartum Psychosis, and the impact that it has had on their lives and relationships.

**Methods:**

Participants (*N* = 8) were partners recruited through the charity Action on Postpartum Psychosis. Partners completed an in-depth, semi-structured interview regarding their experiences of Postpartum Psychosis. Interpretative Phenomenological Analysis was used to analyse the interview transcripts.

**Results:**

Seven superordinate themes emerged from the interview data: loss; powerlessness; united vs. individual coping; hypothesising and hindsight; barriers to accessing care and unmet needs; managing multiple roles; and positive changes from Postpartum Psychosis.

**Conclusions:**

These findings provide a rich illustration of the experiences of partners, including some previously unidentified findings relating to partner hypervigilance to signs of relapse and positive changes in their attitudes and relationships. Areas where support could be provided for partners are also highlighted.

**Electronic supplementary material:**

The online version of this article (10.1186/s12884-018-2055-z) contains supplementary material, which is available to authorized users.

## Background

Postpartum Psychosis (PP) is a severe mental health condition, with a psychotic element and associated mood disturbance following childbirth, and is often described as a ‘psychiatric emergency’ [1, 2]. Postpartum Psychosis occurs in 1 to 2 per 1000 births, with a peak window of onset within the first two weeks after birth [3]. The onset of PP is typically sudden, unexpected and severe [4, 5]. As an emerging field of research, studies have primarily focused on identifying incidence rates, aetiology, exploring genetic susceptibility and risk factors [6–10]. Risk factors that have been identified include having a pre-existing mental health problem (such as Bipolar Disorder), primiparity, marital conflict, lack of social support, and the presence of stressful life events.

The London School of Economics report an 8.1 billion pound spend to cover the direct and indirect costs of perinatal mental health for each annual birth cohort within the United Kingdom [11]. Current National Institute for Health and Care Excellence (NICE) guidance on antenatal and postnatal mental healthcare, recommend that healthcare professionals be alert to any possible symptoms of PP within the first two weeks after childbirth [12]. However, only half of women have access to specialist perinatal mental health services, and both personal and service level barriers to receiving appropriate care have been outlined [11]. Other NICE recommendations include for mental healthcare providers to: establish a co-ordinated care plan; provide medical and therapeutic interventions; and consider the support role of the partner, and the potential effect of the perinatal episode on the couple relationship.

Pregnancy and childbirth are times of considerable change, placing increased demands on a couple. The relationship that a couple have with each other, and the relationship that they build with their infant during the postnatal window, helps to provide a foundation for infant development and secure attachment [13–15]. Establishing this foundation can be difficult in the best of circumstances, but perinatal mental health problems cause an additional challenge. The treatment of, and the course of recovery from, PP has been described as ‘a long and difficult process’, often involving a psychiatric admission of the mother to a general psychiatric ward, or admission of the mother and infant to a Mother and Baby Unit (MBU) ([1], p.155). Whilst the understanding of the impact of perinatal mental health problems for the mother might be improving, the literature provides little information on the impact of such difficulties on partners, or on their relationships with their new infant and the mother.

From the limited research that has been conducted to date, partners typically described the support they felt that the mother required, or lacked, and neglected to mention their own needs; instead, they identified that they struggled to ask for help and felt isolated and overwhelmed [16]. Partners were provided with little support or information regarding PP, and wanted greater support and information for themselves [17]. Although Engqvist and Nilsson [18] sought views from both the mothers and their partners on the recovery process from PP, only four out of twenty-four quotes within this article were from partners, and regarded their interpretation of the mother’s needs at different stages, or their needs as a couple, rather than their own individual needs. Partners were included in the work by Wyatt et al. [19] but this study examined the impact of PP on the relationship and used dyadic interviews. Only one published study was identified to date that focused solely on partners’ experiences of PP [20], drawing on interviews with men during their partners admission to an MBU for first episode PP.

The relevance and importance of pursuing further research into PP can be drawn from considering other neighbouring, but distinct, fields of research, namely that relating to Postnatal Depression (PND). Research by Reid and Taylor [21] into PND has already stressed the protective nature of a supportive couple relationship in the development of postnatal mental health difficulties. Moreover, common themes faced by couples experiencing PND have been identified as including a perceived fracturing of the family unit, a sense of loss, and maternal absence [22, 23].

The aim of the current study was to consider the lived experiences of partners of women who have had Postpartum Psychosis, and the impact that it has had on their lives and relationships.

## Methods

Interpretative Phenomenological Analysis (IPA) was utilised in this study as it is a form of qualitative analysis that allows for the exploration of individuals’ lived experiences with the aim of capturing the insider’s perspective, both unique and collective experiences. Through the process of analysis, data is gained from each individual participant and overarching themes emerge as to what it can be like for someone to go through such an event. This study utilised IPA to explore the lived experiences of partners of women who had been diagnosed with PP as it is an analytic process which allows for both the meaning that partners have made of their experiences to be identified, but also allows for reflection on their affective responses.

The study aim was addressed through semi-structured interviews with partners. In addition, a short online questionnaire was devised and completed at the recruitment stage in order to identify socio-demographic details. Interpretative Phenomenological Analysis (IPA) was applied to the interview transcripts to try to develop an in-depth exploration of partners’ experiences of PP [24]. The use of IPA allows for the identification of some experiences, which may be shared among partners, but also those uniquely expressed. Previous research exploring the impact of Postnatal Depression (PND) on partners has used a similar IPA approach [22].

### Recruitment

The study was given ethical approval from Cardiff University (EC.14.11.11.3914R2A). Participants were recruited through the UK based charity Action on Postpartum Psychosis (APP www.app-network.org). Permission to recruit through APP was obtained from the charity’s director and leadership team. An advertisement for the current research, featuring a brief description of the study and call for participants, was posted on the APP forum website, Facebook page, and Twitter feed. The advertisement included a link to more information regarding the study, a consent form, and access to an online questionnaire for partners to complete and to register if they wished to be contacted with more information regarding an interview. To be eligible for the interview the episode of PP was to have occurred more than 6 months ago but less than 10 years ago, to try and limit emotional distress and for recollection purposes. A further consent form was completed in writing immediately prior to an interview about their experiences of PP.

### Sample

A sample size between 4 to10 is deemed adequate for the completion of IPA [24]. A total of nine partners completed the online questionnaire and agreed to be contacted for an interview, of which eight completed the interview. The ninth partner opted to withdraw from the research prior to an interview being arranged due to their other commitments. The questionnaire data from the ninth participant was subsequently excluded from analysis. All partners who volunteered were male, although the study was open to all couples. The most common age bracket for partners was between 30- to 34-years-old (*n* = 4), although ages ranged from 30- to 49-years-old. Most partners were married (*n* = 7), and all noted they were still with the mother (*N* = 8). The partners’ highest level of educational attainment varied, but the majority had either a master’s degree qualification (n = 4), or an undergraduate degree (*n* = 3), with only one partner noting no qualifications. All partners were in full-time work (N = 8). In terms of the episode of PP experienced, most involved the admission of the mother to either a psychiatric ward or a MBU (*n* = 5). The onset of each episode was typically within the first 2 weeks of childbirth (*n* = 7), with partners reporting the duration of the episode varying between 1 and 3 months to over 1 year.

### Data collection

All of the interviews were completed by the first author (NH) between April and June 2015. Partners were interviewed in their homes (*n* = 1), via SKYPE (*n* = 3), or on the telephone (*n* = 4), based on the participant’s preference. Only one of the eight partners interviewed opted for a face-to-face interview, with most noting that it was easier, and felt more private, for them to complete an interview via a video link or on the telephone. A semi-structured interview design was adopted (Additional file 1). At the start of each interview, the partner was invited to describe their experiences of PP. The subsequent interview schedule was led by the participant, but covered topics relating to partner relationships with the mother and infant, role, decision regarding future pregnancies, and coping and support.

All interviews were audio recorded and subsequently transcribed with all potential identifiers removed. Audio recordings of completed interviews were destroyed once transcription was completed. All data associated with the interviews was stored using awarded pseudonyms.

### Data analysis

All interviews were transcribed by NH to help full immersion in the data. A step-by-step procedure for completing IPA outlined by Smith et al. [21] on the lined, numbered interview transcripts was followed (see Table 1).

**Table 1:** Smith et al.’s [21] stages of IPA

Stage	Description of Stage
1	Reading a transcript
2	Initial notation of a transcript
3	Development of emergent subordinate themes
4	Connections across emergent subordinate themes
5	Moving to the next transcript and repeating stages 1–4
6	Looking across transcripts
7	Testing coherence and plausibility of theme structure
8	Developing a narrative of results and a personal reflection

Respecting the ethos of IPA, analysis of the interview data focused on developing an understanding of the meaning that each partner made of their experience of PP. Both Yardley’s [25] and Elliott, Fischer, and Rennie’s [26] criteria for assessing quality in qualitative research were considered throughout to ensure IPA was completed to a high standard. Yardley’s criteria included exploring: sensitivity to the context, commitment and rigour, transparency and coherence, and the impact and importance of the research. Similarly Elliott et al.’s criteria involved: considering one’s own perspective, situating the sample, providing credibility checks, establishing coherence, accomplishing general and specific research tasks, and ensuring the research resonates with readers. Due to the subjective nature of interpretation, the researcher held an awareness of the different hermeneutic levels involved. The researcher kept a reflexive diary of their experiences at each stage of the research, and any personal or prior experiences that they felt were relevant to reflect upon and bracket during the research process. During the analysis stage the researcher separately noted and bracketed any personal thoughts in order to concentrate on the partner’s own interpretation. After all transcripts had been separately analysed, similarities or differences in themes communicated across partners were identified. The researcher formed an overarching summary depicting a list of superordinate themes across partners. To test coherence and plausibility of the emergent theme structure, the researcher arranged separate meetings with the other research team members, and the charity APP, to discuss the identified themes.

## Results

Seven superordinate themes and 20 subordinate themes were identified across the eight interview transcripts depicting partners’ experiences of PP (see Table 2):

**Table 2:** Theme structure

Superordinate Themes	Subordinate Themes
Loss	Expectation and Loss
	Loss within Couple Relationship
	Trauma
	Life Stops
Powerlessness	Control and Exclusion
	Overwhelming Uncertainty and Unexpectedness
United vs. Individual Coping	Coping Strategies
	Questioning Own Limits
	Couple Unity
	Support and Recovery
Hypothesising and Hindsight	Theorising and Meaning Making
	Guilt and Regret
Barriers to Accessing Care and Unmet Needs	Unrecognised and Unmet Care Needs
	Lack Continuity in Care
	Partner Unmet Support Needs
	Calls for Change
Managing Multiple Roles	Role Alteration
	Neglecting Own Needs
Positive Changes from Postpartum Psychosis	Positives Noted
	Relationship Changes

### Loss

Loss was strongly communicated by partners: from a loss of what was expected in having a child to loss of the couple relationship; physical loss or separation due to hospital admissions; and a feeling that life stops.

You would think that the first time after having a baby you go round obviously showing her off to everybody, and we couldn’t do that purely because [the mother] wasn’t there. *(James*)

At a lot of times, we would go up to [MBU] in the car and I’d go “who is she”[the mother], and I suppose those were the most difficult times. (Mark)

Essentially, I had gained a child on one day and lost my partner on the next day. (Henry)

…during it all there was no husband and wife relationship, which we had been having you know a week or so before [infant] was born. (Peter)

Partners used quite visceral language to refer to their experience of loss, which emphasised the physical and emotional pain felt due to being separated from the mother and infant.

You can’t get a cuddle, that sort of closeness is ripped apart…I felt I had lost not only my wife but my little one, he was in the MBU. (Ben)

A subordinate theme of trauma emerged due to the perceived threatened loss of the mother through PP. Partners described PP as a traumatic experience, stressing their feelings of loss for both the mother and infant. The grief and sense of abandonment experienced by partners is manifest by the frequent use of the word ‘lost’ across the interviews.

I didn’t recognise her at all… it just wasn’t her, but it was almost like she was kind of possessed. I was just terrified really that she... that I had lost her really. (David)

There were times there where we felt, I felt, that we had lost all of our family, that I wasn’t going to have [the mother]. (Ben)

### Powerlessness

Partners noted a sense of powerlessness, encompassing a general lack of control and uncertainty stemming from the birth and ongoing throughout the mother’s PP episode. There was a sense of partners not knowing what was wrong, or how to help the mother, or whether the mother would get better.

But I had no control on the situation, no input into it. I was just holding the sick bowl. (Henry)

I didn’t quite know what was going to happen next really. I think even with my mum and dad, because we just didn’t know what was going on. We didn’t know how to treat it. (Ben)

The language that partners used to describe their experiences highlighted their feelings of being overwhelmed, excluded, and the perceived gravity and enormity of their experience.

I don’t think I could have coped much longer… I honestly don’t think I could have survived much longer [without MBU]. (Ben)

Well it was pretty terrifying really. (David)

Most partners described the unexpected and sudden onset of PP, with the mother having had positive pregnancy experiences up until that point. The language used by partners stressed the severity and swift escalation of PP.

…very rapidly. I mean, thinking back at it, it happened within an hour, or hour and half, of [son] being born. (Stuart)

So, it was all a bit… exploding all of a sudden. (John)

Partners also emphasised their sense of exclusion and not being heard by healthcare professionals, further contributing to their feelings of powerlessness.

…as a partner, I wasn’t allowed to know what was happening…(Henry)

### United vs. individual coping

Different coping styles were adopted; some partners clearly communicated a unified approach to coping as a couple, whereas for others, coping occurred as a separate and dividing process between the partner and the mother.

…we thought something was wrong but we didn’t know what. (John)

[To the mother] “Look, you have to snap out of things, and if you don’t I’m out of here because I can’t cope with this”. (Henry)

A variety of individualised coping strategies were identified by partners. Some partners focused on practicalities (such as care routines), or pursuing normality, or taking an approach of planning one day at a time. Others had to learn to talk about their emotions, to speak as a couple; and some found dry humour to be helpful. Some partners reported needing their own space (so took the dog for a walk), or found it useful to vent to friends. For others, partners needed to seek out other people who had experienced PP, or search for information on PP. Utilising familiar work-stress management strategies were adopted by some, as was attributing blame (for example, believing that the mother’s birth plan choices resulted in the episode). All partners questioned their ability to cope and sought support, even if that was anonymised support through an online forum. Partners noted it was ‘impossible’ (David) to comprehend how they would have coped without familial support.

The thought of being a father was going to be tough and then you have that on top of it. Your world comes crashing down all around you. Looking back now you kind of think I don’t know how I got through that. (Mark)

Coping often involved partners having to become a ‘specialist’ (Mark) in PP in order to understand what was happening to the mother, and answer questions that healthcare professionals had failed to address. The word ‘specialist’ demonstrated the need to self-educate about the nature and development of PP, to become expert in order for partners to better cope and understand the experience. Furthermore, it implied a lack of communication and information from healthcare professionals, which motivated partners to commence a process of information seeking and assimilation, in order to generate answers to partners’ outstanding queries regarding PP. Partners often referred to needing to research PP but the language that Mark used captured the extent to which partners can adopt information gathering as a means to better cope with their experience.

I’m now a specialist in postpartum psychosis medication involved, because I am one of those people who will research in terms of Google and APP networks and what not. I guess that had been my idea of finding out what is actually going on. (Mark)

Issues that partners highlighted encountering when trying to cope included: having to compromise their values; having no opportunity to vent their emotions and to prioritise the mother’s needs; and continuing to question the mother’s behaviour (including her humour or signs of energy) after recovery for fear the mother was becoming unwell again.

She had to stop breastfeeding which she didn’t want to do… (Ben)

…I analyse our conversations more than I should really. (James)

Partners although praising the support provided by families, also reported the difficulties in setting boundaries for extended family involvement in providing care.

I think it strained… because the in-laws came and lived over at the house for a bit and helped out quite a bit. Which was good but hard to tell them to back off. (John)

Partners noted that generally they were not offered any support from healthcare professionals, and when it was offered struggled to understand the nature of the support and failed to believe any reassurances given.

I didn’t know what sort of support I needed. In fact I would say they didn’t help… I was completely lost as to what a social worker does and what benefit it was to me. (Henry)

…I thought she [nurse] was trying to comfort me rather than it actually being true…it was pretty hard, impossible to believe really at the time. (David)

### Hypothesising and hindsight

Partners typically developed a specific theory about what they felt may have triggered the mother’s episode of PP. These hypothesised triggers differed across partners and included postnatal difficulties such as poor sleep, exhaustion, pressures or problems breastfeeding, and pressures relating to feeling unable to following a child rearing book. Other hypothesised triggers related to the birthing experience, such as, experiencing a difficult labour, unexpected changes to the birth plan, and or developing a sense of loss of control during the birthing process. Physiological changes due to labour were also considered by partners to be potential triggers, such as hormonal changes and anaemia.

Finally, delays in appropriate treatment were considered as significant in the further development of symptoms, for example, delayed access to appropriate perinatal mental health services and mothers being prescribed antidepressant medication which appeared to worsen symptoms. The benefit of reflection was remarked upon, especially as a couple, in making sense of their experiences. James reflected upon their infant as ‘this little person’, highlighting the sense of reality, responsibility and vulnerability faced by partners in trying to adequately meet their infants’ care needs in the context of also trying to care for the mother.

…we feel that the sleeplessness was very much a big thing… we have actually got this little person to look after…. *(James*)

Partners also communicated feelings of guilt and regret, often commenting on things they felt they ‘should have’ done.

…there is a huge amount of guilt I felt that I had pushed her into going out and things like that… because I’d been told to. But actually that didn’t help her at all and I felt masses amount of guilt. (Ben)

I suppose just feeling negative… that I let [the mother] down, when I should have been…[partner paused]. (Stuart)

…I probably felt if anything guilty that I wasn’t there in the Unit. (David)

A final regret reported by partners was that of not being provided with information to make an informed decision as to whether to have a second child, and thus opting not to.

…it would have changed my mind, having read other stories, evidence and stuff, I think we would have been in a better position to do that [decision to not have a second child]. (Ben)

### Barriers to accessing care and unmet needs

Partners identified barriers to accessing care for the mother. These included healthcare professionals failing to accurately identify what was wrong with the mother due to poor understanding, the lack of empathy from primary care services, and the lack of consistency in care and communication between healthcare professionals.

…[GP] just told [the mother] to go and have a bath, try and chill out for a bit, and take some natural sleeping remedies and you’ll be fine…that’s another day or two down the line and we tried that and she just got worse and worse and worse. (James)

…people in the healthcare profession, they could have helped more, and got to get us support sooner, that is at the centre of everything. (Stuart)

…the locum at the end was “you’ll have to try harder than that to kill yourself”, and I could have disappeared I was so angry. (Ben)

Partners described not being heard, either individually or as a couple, by healthcare professionals, and if they were listened to then it was only after the mother had reached crisis point. For most partners, no enquiry was made by healthcare professionals to the partner’s own needs and no support offered.

…when the medical teams would be coming around and I would be talking to them, and thinking back, ignoring every single word I said…From the typical bloke perspective it was just kind of assumed by everyone that everything would be okay. There was never much discussion, even from family, but certainly not from any of the medical services, as to how I was, what concerns did I have. I was very much left to feel that you have got to cope… [this interview] …is probably the first time I actually had the opportunity to express it because going right back to day one no one ever really asks. (Peter)

Partners advocated that some changes need to happen, for instance: increasing the number of local MBUs, having noted them to be a ‘lifesaver’ (Mark); raising awareness of PP among primary care staff; educating partners about PP and increasing the support offered to them; and helping couples to be aware of PP and feel prepared for it, as this had helped some partners following later pregnancies.

The whole MBU staff was fantastic because they know it, and I think, at that point that was the first thing I’d heard of postpartum psychosis. (Ben)

…those places [MBU] are a lifesaver, because if you are not in that specialism no one else can really understand. (Mark)

### Managing multiple roles

Partners commented on the conflict between their many new roles and meeting the care needs of their family, particularly in the context of trying to maintain a job. In order to manage, partners would typically prioritise the mother’s, or infant’s, needs above their own, and would often then have to prioritise whether to care for the mother or their infant.

…I still had to work, there was no one who could cover my job, and look after a newborn, not getting much sleep, my wife is potentially on suicide watch, and being watched 24/7…There wasn’t really time to stop and think how am I feeling because there was no choice but to deal with everything. (Peter)

…I felt I struggled to sleep, because I wanted to make sure that she was sleeping and that put me on edge and I felt very agitated myself. (James)

Partners noted a shift in their couple relationship. David’s quote signified a shift in the partner's role within the couple relationship, from one of mutual supporter to that of protector and carer. The perceived vulnerability of the mothers by partners altered the relationship dynamic, and for some resulted in adopting a modified, non-egalitarian and paternalistic communication style.

…it was almost like speaking to a child I guess in a way… (David)

### Positive changes from postpartum psychosis

The majority of partners detailed a number of personal positive changes that had occurred due to PP, including increasing partners’ own empathy and understanding towards mental health issues in general.

…it gave us an empathy, not even with PP… people going through depression… I was probably being a bloke’s bloke beforehand in terms of thinking about people with depression. “Just snap out of it” type, sort of thing. (Ben)

I think I am far more aware of how [the mother], or how I perceive [the mother] to be feeling, so even without her saying anything I will find myself second-guessing how I think she is feeling… (Peter)

Furthermore, there was recognition for MBUs assistance in establishing a care routine for their infant.

…If there was any silver lining to be had out of this whole story, it was that the Mother and Baby Unit taught you how to look after a little one. (Ben)

Positive changes to the couple relationship were also noted by some partners. Following the episode of PP, some couple relationships had reportedly strengthened and became more supportive. Moreover, partners had experienced more time to bond with their infant, and had concentrated on redressing their work-life priorities.

I’ve always said that it has got better. It’s got stronger, that sounds really odd… I always say that we are actually stronger for it. These things make you feel stronger, I guess…My focus used to be very different. I used to be quite career driven… whereas now I’m like it’s just get through the day, get home. I use to work 90% of this time. Whereas now I’m out the door at one minute past five. (Mark)

…we are both very supportive of each other in life… when one seems exhausted and drained then we give each other a break, but then we kind of more sympathetic than before. (David)

## Discussion

The aim of the study was to develop an understanding of the lived experiences of partners of woman with Postpartum Psychosis (PP). Seven themes were identified: loss; powerlessness; united vs. individual coping; hypothesising and hindsight; barriers to accessing care and unmet needs; managing multiple roles; and positive changes arising from PP.

Several of the themes were comparable to those found in perinatal mental health research. The sense of loss communicated across many dimensions including loss of the expected future echoed the findings with mothers who had experienced PP [1] and reflects the work on expectation-loss theory of what might, or what could, have been [27]. Themes identified in studies exploring partners’ experience of PND [22, 23] also centred on loss including loss of control, intimacy, expectation and routine, alongside feelings of helplessness in caring for their family and the “fracturing” ([22], p717) of the family unit.

The powerful individual narratives and the themes that have emerged underline the impact of PP on the partners and further evidence the need for implementation of the NICE recommendations for mental health providers to consider the needs of the partner and the effect of PP on the couple relationship [12]. Women who have experienced PP recognise that their partners need access to psychological support [1]. However, partners in this study neglected their own needs (similar to previous findings [16]), so without the healthcare professionals being alert to this it will be difficult to reduce the known risk to partners mental health when a woman has postnatal mental health problems [28–30]. This is an important consideration for the well-being of all members of the family unit including the infant; the partners in this study took on a greater caregiving role for the infant than they had planned and research suggests that partners take on a buffering role in the development of attachment in their infant, in the context of postnatal mental health problems [31, 32]. Interestingly the positive changes described by some partners as a result of PP often related to this shift in role, having more time to bond with their infant and changing priorities away from work to prioritising time with their family.

Partners described a range of coping strategies. Alongside well recognised strategies previously identified in this context [28] such of seeking normality, and trying to source information, there were other strategies which required couples to compromise on their values in order to cope, for example, making the decision for the mother not to breastfeed in order to receive medication. This type of compromise appears common when there are postnatal mental health problems; unfulfilled dreams or wishes was a theme extracted from narratives of women who had experienced PP [33]. In order to try and make sense of their experience many partners developed their own theory about the trigger for the PP. Some included those postulated within the existing research literature, for example, sleep loss, or antidepressant medication resulting in mania [9, 34] but others were quite specific and particular to their family circumstances.

The benefit of familial support was clearly communicated by partners, noting that it would have been ‘impossible’ to comprehend coping without such support, echoing the views of women who experienced PP [1]. However, some partners identified the need to establish boundaries around familial support, and at times felt the need for families to step back. This has not previously been noted within the research literature, but may link to the sense of powerlessness that partners report during the episode of PP, and their need to feel in some way in control of the situation.

Significant expectation was placed on partners, by both family members and healthcare professionals, to provide support. However, they felt that their concerns were unheard and they were largely excluded from the mother’s care and treatment which exacerbated the sense of powerlessness and uncertainty. The lack of clear consistent communication from the healthcare professionals had several consequences. The importance of reassurance by healthcare professionals has previously been identified from research with mothers [35] but the partners in this study reported that they struggled to believe any of the reassurances, due to previous inaccurate information about the mother’s condition. Similarly they found it difficult to trust the recovery process as they continued ‘second guessing’ whether the mother would become unwell again, misinterpreting humour, or energy, as a sign of mania or psychosis returning. This finding has not previously been identified within the PP research literature, but links with the change in the relationship dynamic and a potential shift in the partner’s frame of reference by which they interpret and understand the mother’s actions which may endure after the mother is considered recovered.

Partners identified a number of barriers to care and unmet needs, many of which indicated a failure of services to adhere to the gold standard described in the NICE guidelines [12], and reflected the lack of access in the UK to perinatal mental healthcare services [11]. The requirement for improvements in perinatal mental health service provision was recognised in the Chief Medical Officer’s annual report in England, and the Together for Mental Health Delivery Plan 2016–2019 in Wales [36, 37] so services may have improved in response. However, based on these findings, the implications for practice broadly fall into three domains: types of service provision, improved knowledge and communication both between healthcare professionals and between the professionals and family. Those who had access to an MBU clearly valued the service so broader availability of this type of service is a priority. There needs to be improvements in knowledge about PP and its management in primary and secondary care in order to improve the quality and accuracy of communication. Continuity of care would be improved by an assigned care co-ordinator acting as a point of reference for partners and other healthcare professionals. Partners play such a significant role in PP that any education package for healthcare professionals must underline the need to involve them, listen to them but also to be alert to their mental health needs as well. Campaigns targeted at parents-to-be to inform them of signs of perinatal mental health difficulties and how to seek help could also help to raise awareness in the population.

There is a growing literature on partners’ experiences in the context of perinatal mental health but this exploration of partners’ own experiences of PP is largely unique at this time. The qualitative design of the current study is a strength as it attempts to maintain the voice and personal experiences of partners. It has led to findings which fit with the existing research in areas such as PND but also advances our understanding of the experience of PP and therefore offers information to inform improvements in practice.

One potential limitation with regards to data analysis is that all initial coding was completed one researcher, however, criteria for assessing quality in qualitative research was considered throughout to try to ensure that IPA was completed to a high standard. The approach to sampling was appropriate to the method but as the partners were all recruited through a charity other potential participants would have been missed and it might also be the case that allowing for an upper time-frame of 10 years could have had an impact on recall. The fact that all couples were still together could potentially have biased the reporting of changes in the couple relationship as it is known that there are high rates of separation and divorce following postnatal mental health problems [38]. Some of the themes extracted that relate to partner experiences could be argued to represent the experiences of new fathers, without the context of PP; qualitative research with first time fathers [39] suggests there is some overlap, for example, in the theme of helplessness mirroring to some degree the theme of powerlessness, but many of the other themes appear to denote more about the practical demands placed on new fathers, such as the theme of trial and error parenting.

## Conclusions

We believe the current study will help develop a more thorough understanding of PP and the impact that it can have on partners and the couple relationship. In order to support partners, services need to be aware of and responsive to their experiences which may include loss and trauma, emotional responses (such as feelings of guilt, regret and self-blame), the multiple roles they are managing, and the impact on the couple relationship. The study has also helped to identify current barriers to care and unmet needs, from a lack of awareness of PP and delays in accessing appropriate treatment, to a lack of support for, or consideration of, partners. Overall, the impact of PP on partners is broad and substantial and requires consideration by healthcare professionals in order to secure the best clinical outcomes for all members of the new family unit.

## Additional file

Additional file 1: Interview Topic Guide. (DOCX 13 kb)

## Abbreviations

APP: Action on Postpartum Psychosis
IPA: Interpretative Phenomenological Analysis
MBU: Mother and Baby Unit
PND: Postnatal Depression
PP: Postpartum Psychosis
