# Supplementary material for: The impact of postpartum psychosis on partners
Source: BMC Pregnancy Childbirth. 2018 Oct 23;18:414. doi: 10.1186/s12884-018-2055-z (PMC6199718; doi:10.1186/s12884-018-2055-z)
Supplement: Supplementary file 1 — Interview Topic Guide. (DOCX 13 kb) [file 12884_2018_2055_MOESM1_ESM.docx]

Interview Topic Guide

1. Tell me about your experiences of PP? What happened?
2. What do you feel triggered the episode?
3. How did you cope?
4. What did a typical day look like to you at that time?
5. If you compared your role and responsibilities within your immediate family during that episode to before the episode would it look different? If so how?
6. If you compared your role and responsibilities within your immediate family during that episode to now, would it look different? If so how?
7. Do you feel it impacted on your relationship with your partner at the time? If so, how? What about now?
8. Do you feel your communication style with your partner changed during the episode of PP, or since?
9. Do you feel your sexual relationship with your partner changed during your partner’s recovery, or since?
10. Do you feel it impacted on your relationship with your child at the time? If so, how? What about now?
11. Do you have any other children? Do you feel it impacted on your relationship with your other child/children? If so, how? What about now?
12. Do you feel the episode of PP effected your decision to have more children? How did you and your partner make this decision? Do you feel this decision making process impacted on your relationship with your partner?
13. Do you feel if impacted on other areas of your life at the time? If so, how? What about now?
14. Do you feel it impacted on other relationships you had at the time? E.g. family, friends, work colleagues?
15. Do you feel there have been any enduring effects from PP?
16. Looking back, what things do you feel would have helped at the time? Any unmet needs?
